# Gentle Rhodamines for Live-Cell Fluorescence Microscopy

**DOI:** 10.1021/acscentsci.4c00616

**Published:** 2024-10-02

**Authors:** Tianyan Liu, Julian Kompa, Jing Ling, Nicolas Lardon, Yuan Zhang, Jingting Chen, Luc Reymond, Peng Chen, Mai Tran, Zhongtian Yang, Haolin Zhang, Yitong Liu, Stefan Pitsch, Peng Zou, Lu Wang, Kai Johnsson, Zhixing Chen

**Affiliations:** † College of Future Technology, Institute of Molecular Medicine, National Biomedical Imaging Center, Beijing Key Laboratory of Cardiometabolic Molecular Medicine, Peking University, Beijing 100871, China; ‡ Peking-Tsinghua Center for Life Science, Academy for Advanced Interdisciplinary Studies, State Key Laboratory of Membrane Biology, Peking University, Beijing 100871, China; § Department of Chemical Biology, Max Planck Institute for Medical Research, Heidelberg 69120, Germany; ∥ Biomolecular Screening Facility, École Polytechnique Fédérale de Lausanne (EPFL), Lausanne 1015, Switzerland; ⊥ PKU-Nanjing Institute of Translational Medicine, Nanjing 211800, China; # GenVivo Tech, Nanjing 211800, China; ∇ Spirochrome AG, Chalberwiedstrasse 4, CH-8260 Stein am Rhein, Switzerland; • College of Chemistry and Molecular Engineering, Synthetic and Functional Biomolecules Center, Beijing National Laboratory for Molecular Sciences, Key Laboratory of Bioorganic Chemistry and Molecular Engineering of the Ministry of Education, PKU-IDG/McGovern Institute for Brain Research, Peking University, Beijing 100871, China; ° Key Laboratory of Smart Drug Delivery, Ministry of Education, School of Pharmacy, Fudan University, 201203 Shanghai, China

## Abstract

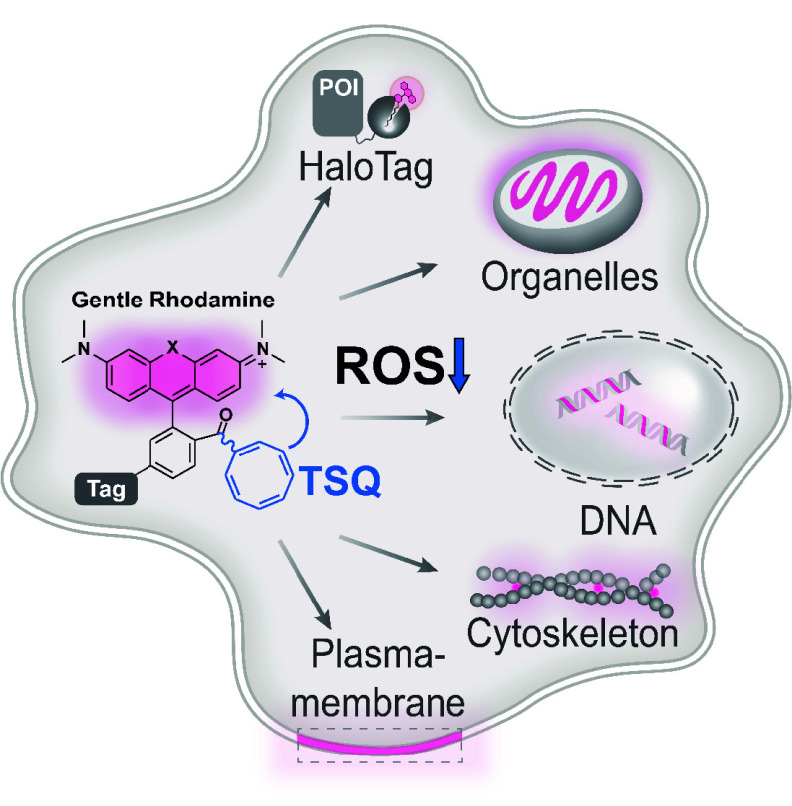

Rhodamines have been continuously optimized in brightness, biocompatibility, and color to fulfill the demands of modern bioimaging. However, the problem of phototoxicity caused by the excited fluorophore under long-term illumination has been largely neglected, hampering their use in time-lapse imaging. Here we introduce cyclooctatetraene (COT) conjugated rhodamines that span the visible spectrum and exhibit significantly reduced phototoxicity. We identified a general strategy for the generation of Gentle Rhodamines, which preserved their outstanding spectroscopic properties and cell permeability while showing an efficient reduction of singlet-oxygen formation and diminished cellular photodamage. Paradoxically, their photobleaching kinetics do not go hand in hand with reduced phototoxicity. By combining COT-conjugated spirocyclization motifs with targeting moieties, these Gentle Rhodamines compose a toolkit for time-lapse imaging of mitochondria, DNA, and actin, and synergize with covalent and exchangeable HaloTag labeling of cellular proteins with less photodamage than their commonly used precursors. Taken together, the Gentle Rhodamines generally offer alleviated phototoxicity and allow advanced video recording applications, including voltage imaging.

## Abstract 2

Phototoxicity is an open challenge in live-cell microscopy. Gentle Rhodamine probes reduce the formation of reactive oxygen species (ROS) and enable long time-lapse imaging of different organelles.

## Introduction

Modern fluorescence microscopy has evolved from 3D imaging of fixed specimens to 4D recording of subcellular structures or dynamic cellular processes in live cells or animals. Herein, spatial resolution beyond the diffraction barrier and temporal resolution of video rates can be achieved.^1−3^ However, time-lapse recording at high resolution subjects the live samples to significantly elevated light doses, surpassing orders of magnitude the levels employed in typical one-shot wide-field or confocal imaging experiments.^4^ High excitation light exposure of fluorescent labels is known to compromise the physiological integrity of biological samples.^5−7^ This phenomenon, referred to as phototoxicity, is the reversible or irreversible damaging effects of light and fluorophores on its surroundings. Phototoxicity mainly originates from reactive oxygen species (ROS), which are generated by the excited states of chromophores.^6−8^ A major ROS relevant to phototoxicity in live-cell fluorescence microscopy is singlet oxygen, which is the product derived from the reaction of the excited fluorophore and molecular oxygen.^9−11^ The reactive singlet oxygen can oxidize nearby biomacromolecules such as lipids, carbohydrates, and nucleic acids, thereby affecting their physiological functions.^11−15^ Such harmful effects accumulate over time and result in abnormal cell metabolism, deformation of organelles and organisms, arrested cell proliferation, and apoptosis.^7,16−18^ Therefore, phototoxicity is a universal phenomenon that widely affects live-cell fluorescence imaging practice, rendering it potentially invasive. With the democratization of super-resolution imaging and time-lapse imaging instruments, minimizing phototoxicity is of growing importance to endorse the physiological relevance of the recorded data in bioimaging.

**Figure 1:**
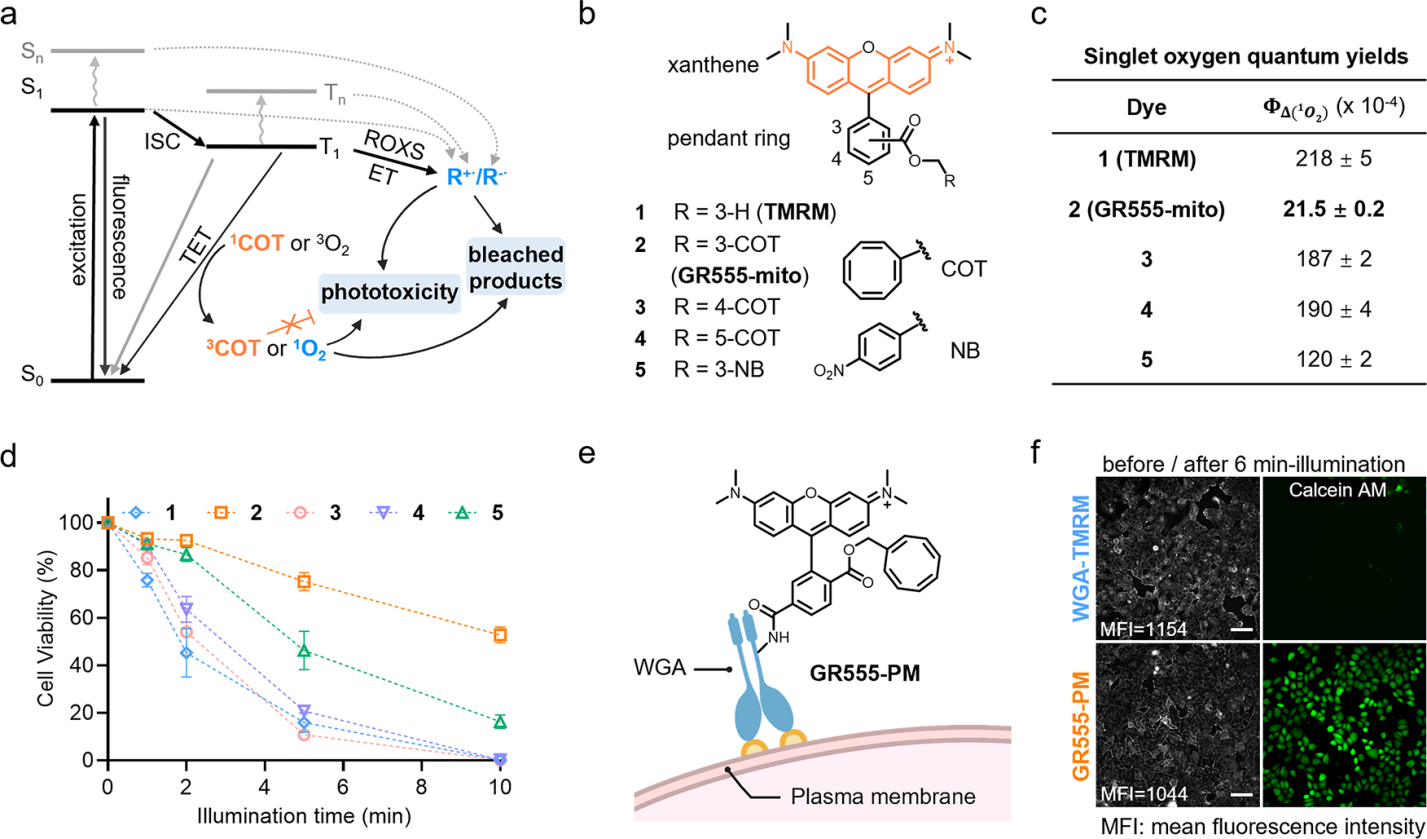
**Derivatizing TMR with TSQ alleviates phototoxicity.** (a) Jablonski diagram depicting different ways of relaxation from the exited state S_1_ including photobleaching, phototoxicity, and triplet-state quenching. ISC: Intersystem crossing; TET: Triplet-energy transfer; ROXS: reducing and oxidizing system; ET: Energy transfer. (b) Chemical structures of tetramethyl rhodamine-TSQs (TMR-TSQs) probes. (c) Absolute singlet oxygen quantum yields (Φ_Δ_) of compounds **1**–**5** under 520–530 nm LED light irradiation. The decay slope of DPBF shown in Supplementary S3d is positively correlated with the singlet oxygen quantum yield. Standard deviations of three independent repeats. (d) Live-cell phototoxicity measurements of compounds **1**–**5** (250 nM, 15 min) in HeLa cells. Cell apoptosis of >500 cells after 561 nm LED light illumination (1.4 W/cm^2^) was examined at each time point of the three independent experiments. Error bars indicate the standard deviation. (e) Schematic representation of a low-phototoxic probe, **GR555-PM**, for plasma membrane labeling. (f) Live-cell images of HeLa cells labeled with **WGA-TMRM** (30 μg/mL, 5 min) or **GR555-PM** (50 μg/mL, 5 min) (gray) and stained with Calcein AM (1 μM, 5 min, green) after illumination of 532 nm LED (∼2.6 W/cm^2^) at different illumination time. Scale bars = 100 μm. MFI: the mean fluorescence of intensity.

From the perspective of photophysical chemistry, phototoxicity, and photobleaching are generally believed to stem from the excited triplet states (Figure 1a). Triplet state quenchers (TSQs), such as mercaptoethylamine (MEA) or cyclooctatetraene (COT), have a rich history of serving as protective agents in live-cell fluorescence imaging.^11,19−23^ In the past decade, the direct conjugation of such TSQ moiety on a selected dye scaffold has been proposed as a strategy for increasing photostability,^19,20^ particularly demonstrated with single-molecule imaging using cyanine dyes. Recently, our laboratory and others have repurposed these photophysically sophisticated molecules for live-cell super-resolution imaging of mitochondria and plasma membrane-voltage imaging,^11,21−24^ where phototoxicity has emerged as a complementary threat to photobleaching. These pioneering works are niche demonstrations tailored for a small number of cellular structures using cyanine or fluorescein dyes, whose charged chemical nature limits their general biological applications.

Rhodamine dyes featuring excellent photophysical properties, spectral tunability, and cell permeability, have dominated the field of live-cell fluorescent imaging in recent years. Particularly, silicon rhodamine-(SiR)^25^-carboxyl-based probes (SiR-actin,^26^ SiR-tubulin,^26^ and SiR-Hoechst^27^) possessing fluorogenicity^25,28^ due to an environmentally sensitive equilibrium between a fluorescent zwitterion and a nonfluorescent spirolactone, helped to increase the cell-permeability and reduce the background fluorescence in live-cell imaging. In addition, a general strategy tuning this dynamic equilibrium by introducing (sulfon) amide modifications to the 3-carboxylic acid was subsequently established to create multicolor fluorogenic rhodamines for live-cell nanoscopy.^29^ To meet the growing demands of fluorescence nanoscopy, the photophysical properties of dyes such as brightness,^30,31^ photostability,^32−35^ and blinking^36,37^ have been further engineered. Most importantly, rhodamines can be used in conjunction with a variety of labeling techniques for instance self-labeling protein tags (HaloTag,^38^ SNAP-tag,^39,40^ and TMP-tag^41^), tetrazine for click chemistry,^42^ biomolecular ligands^26^ and specific ligands for organelles.^26,27^ This synergistic development has also consolidated rhodamine as a mainstream tool for bioimaging. Along the line of photophysics, the Blanchard group made a first attempt on silicon rhodamine-COT conjugate using a C6 linker, which gives a marginal increase of photostability.^43^ From the phototoxicity perspective, the systematic upgrading of rhodamines toward reduced ROS generation would lead to another breakthrough for imaging tools in the field of 4D fluorescence imaging.^18^

Here, we introduce a general TSQ-conjugation strategy to reduce the phototoxicity of rhodamine derivatives. As confirmed by *in vitro* singlet-oxygen production or protein damage assays, as well as live-cell phototoxicity studies to various subcellular compartments, Gentle Rhodamines (GR) are valuable tools for light-intense microscopy applications. Interestingly, the TSQ-rhodamine derivatives do not necessarily bear enhanced photostability, implying alternative photobleaching pathways that are independent of triplet state populations or include higher excited states.^33,44^ This strategy is compatible with a broad range of fluorogenic rhodamine derivatives and popular live-cell labeling strategies, such as self-labeling tags or ligands for subcellular structures, bringing out a practical dye palette for general and gentle imaging of mitochondria, plasma membrane, nucleus, cytoskeletons, and proteins of interest in mammalian cells. We demonstrate that GR probes can be combined with microscopy techniques like time-resolved STED and functional imaging of the membrane potential in cardiomyocytes.

## Results

### Generation of Gentle Tetramethyl Rhodamine through COT Conjugation at the 3-Carboxyl Position

To systematically profile the structure–activity relationship of rhodamine-TSQ conjugates, we selected COT and nitrobenzene as representative TSQs and synthesized their rhodamine derivatives. Cyclooctatetraene-1-methanol was conjugated to tetramethyl rhodamine (TMR) at different positions on its lower pendant ring, resulting in compounds **2**–**4** and nitrobenzyl alcohol was coupled to 3-carboxy TMR to create compound **5** (Figure 1b and Supplementary Figure S1a-e, see Method for details). It has been previously demonstrated that TSQs affect the photophysics of Cy5 in a distance-dependent manner.^43^ X-ray crystallography analysis of compound **2** highlights the proximity between the xanthene chromophore and the COT moiety (Supplementary Figure S2). Notably, the COT moiety is conformationally flexible as evident from the two components in the single crystal, further enhancing its effective collision with the chromophore.

We evaluated the TSQ-TMR conjugates and the reference compound TMR methyl ester (**TMRM**, compound **1**) from three aspects: (i) photostability; (ii) *in vitro* singlet oxygen generation; and (iii) phototoxicity based on cell apoptosis. First, in organic polymer films mimicking the amphiphilic cellular environment, compounds **1**-**4** displayed high photostability, whereas compound **5**, the nitrobenzene derivative, was more prone to photobleaching (Supplementary Figure S3a,b). Next, all TSQ-conjugated dyes exhibited reduced singlet oxygen generation (Figure 1c and Supplementary Figure S3d and Table S1), as measured by the singlet oxygen-induced decay of 1,3-diphenylisobenzofuran (DPBF) under the illumination of a 520–530 nm LED lamp.^45^ The lowest singlet oxygen quantum yield was measured with the TMR derivative bearing COT in the closest proximity (3-carboxy) to the chromophore (compound **2**, Φ_Δ_: (2.2 ± 0.1) × 10^–3^), which is 10-fold reduced compared to that of **TMRM** (compound **1**, Φ_Δ_: (2.2 ± 0.4) × 10^–2^, Figure 1c, Supplementary Figure S3c, and Table S1). Finally, we stained HeLa cells with compounds **1**–**5** to assess the phototoxicity through a photoinduced apoptosis assay. The positive charge of these dyes leads to a bright fluorescent signal inside mitochondria, an organelle that is vulnerable to photodamage leading to apoptosis. Apoptosis was evaluated using a high-content imager and propidium iodide (PI) staining. The half-lethal light dose for cells stained with the reference compound **1** was reached after only 2 min-illumination with a 561 nm LED lamp. Remarkably, for compound **2**, the dose was reached after 10 min-illumination, while it required 2–5 min-illumination for the TSQ-conjugated compounds **3**–**5** to kill 50% of the cells (Figure 1d). In summary, COT-conjugation at 3-carboxyl of TMR can achieve the largest reduction in phototoxicity among the screened isomers. Such a probe could extend the duration of time-lapse recording by about five times compared to compound **1** without affecting the photostability, wherefore we named this probe Gentle Rhodamine **GR555-mito**.

Having identified **2** as a gentle rhodamine dye, we coupled it to wheat germ agglutinin (WGA) via its 6-carboxyl succinimidyl ester (**S25**, Supplementary Figure S4a), yielding **GR555-PM**, a bright fluorescent marker for plasma membrane of the live cells (Figure 1e,f). We monitored its cellular phototoxicity in comparison to **WGA-TMRM** in a high-content imager by assessing cell viability using Calcein AM stain after continuous imaging of the labeled membranes. Compared to **WGA-TMRM**, our COT-bearing variant **GR555-PM** showed lower cellular phototoxicity by a factor of 4 with a half-lethal dose of 10 min-illumination (Supplementary Figure S4c). This assay confirmed the reduced phototoxicity of COT-conjugated rhodamines, at the same time presenting a practical and gentle membrane stain.

### COT Conjugation Gives Access to Gentle Rhodamines with Diverse Auxochromes in Various Colors

**Figure 2:**
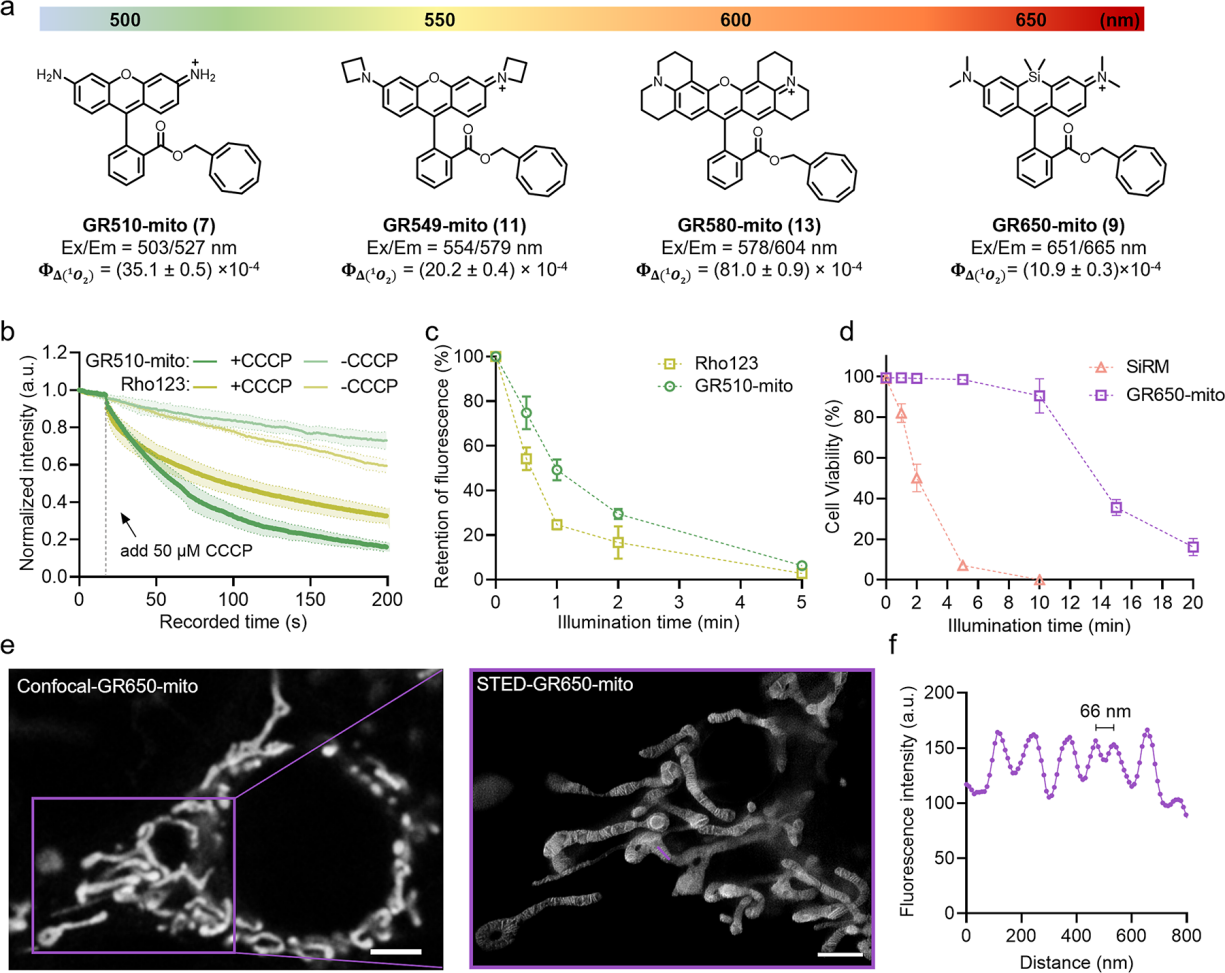
**Multicolor gentle rhodamines for live-cell fluorescence microscopy of mitochondria.** (a) The chemical structures of compounds **7**, **9**, **11**, and **13**, their wavelengths of the maximum absorption and emission peaks and (reduction in) absolute singlet oxygen quantum yields (compared to reference dyes) (see Figures S7 and S8). (b) Normalized fluorescence intensities of time-lapse recordings of COS-7 cells labeled with **Rho123** or **GR510-mito** (300 nM, 60 min) after the addition of carbonyl cyanide 3-chlorophenylhydrazone (CCCP). Control samples were treated with **Rho123** or **GR510-mito** without the addition of CCCP. Data points represent averaged fluorescence intensity curves of six cells from two independent biological replicates. Error bars, showing light-shaded areas, indicate the standard deviation. (c) Phototoxicity of **GR510-mito** or **Rho123** (200 nM, 30 min) in HeLa cells, as measured by the analysis of fluorescence retention in mitochondria after 488 nm LED illumination at different times. Data points indicate the mean of at least 1,500 individual cells from three independent biological replicates after bleaching correction. Error bars indicate the standard deviation. (d) Phototoxicity of **GR650-mito** and **SiRM** (both 250 nM, 15 min) in HeLa cells, measured by cell apoptosis assay after a 640 nm LED illumination (650 nm, 1.2 W/cm^2^) at different time points. Data points indicate the mean of at least 1,500 individual cells from three independent biological replicates. Error bars indicate the standard deviation. (e) Confocal microscopy (left) and STED nanoscopy (right, zoom in) of live COS-7 cells labeled with **GR650-mito** (250 nM) for 15 min at 37 °C. Scale bar = 2 μm. (λ_ex_ = 640 nm, λ_STED_ = 775 nm). (f) Fluorescence intensity line profiles measured as indicated in the magnified view of the purple boxed area in (e).

In order to extend our design to other commonly used rhodamine derivatives, green-emitting Rhodamine 110 and far-red-emitting SiR dyes were esterified with COT-alcohol, giving rise to two novel mitochondrial dyes, **7** (**GR510-mito**) and **9** (**GR650-mito**). Both probes offer similar photobleaching rates and reduced singlet oxygen generation than their methyl-ester counterparts, **6** (**Rho123**) and **8** (**SiRM**) (Figure 2a, Supplementary Figures S5–S7, and Table S1). In addition to TMR, we also esterified JF_549_ bearing azetidine auxochromes and Rhodamine 101 bearing julolidine auxochromes with COT-alcohol (Compound **11**: **GR549-mito** and **13**: **GR580-mito**, Figure 2a and Supplementary Figure S8). Again, the singlet oxygen generation was drastically reduced for both fluorophore scaffolds (Supplementary Figure S7). Overall, COT-conjugation is a general approach to alleviate the phototoxicity of the state-of-the-art rhodamine palette.

We then performed the light-induced apoptosis assay with this set of gentle mitochondrial dyes. Unlike the TMR derivatives which trigger apoptosis under light-illumination, **Rho123** and **GR510-mito** rapidly escaped from mitochondria before the induction of apoptosis, most likely due to a higher hydrophilicity. We first verified that **GR510-mito** is a fast-acting mitochondrial membrane potential (MMP) indicator like **Rho123/TMRE**: Upon addition of an oxidative phosphorylation uncoupler (carbonyl cyanide 3-chlorophenylhydrazone, CCCP), we recorded a rapid fluorescence decrease of **GR510-mito** or **Rho123/TMRE** signals (Figure 2b and Supplementary Figures S9 and S10) reflecting the MMP level and mitochondrial health. Therefore, for **Rho123** and **GR510-mito**, phototoxicity was evaluated by their light-induced decrease in mitochondrial fluorescence after photobleaching correction (Supplementary Figure S11). **GR510-mito** led to 50% cellular MMP reduction after 1 min-illumination with a 488 nm LED, whereas the control compound **Rho123** showed a more rapid decrease of MMP with a half-life of only 0.5 min (Figure 2c). Conversely, the far-red dyes **GR650-mito** and **SiRM**, showed slow mitochondrial leakage, but induced cell death upon long-term illumination. Therefore, we assessed the cellular phototoxicity by monitoring the photoinduced apoptosis. The half-lethal light dose of the cells stained with **GR650-mito** was reached after 10–15 min-illumination with a 640 nm LED, which is 5–7-fold higher than that of **SiRM** (half-lethal at 2 min-illumination) (Figure 2d). These results demonstrated that the COT-conjugation reduces *in vitro* singlet oxygen generation and cellular phototoxicity of rhodamine derivatives with different colors.

As practical mitochondrial stains, cyanine-COT conjugates generally give stronger fluorescence signals than rhodamines (Supplementary Figure S12). Yet **GR510-mito** fills the green to yellow spectrum niche that was not covered by PK Mito probes. Moreover, **GR650-mito** like other SiR-based probes has far-red emission and a similar quantum yield compared to its parent compound **SiRM** (Table S2). We demonstrate the compatibility with commercial STED nanoscopy systems equipped with a 775 nm depletion laser by the visualization of the cristae organization of COS-7 cells, which enabled us to distinguish adjacent crista at the spacing of 66 nm (Figure 2e, f). Notably, unlike **TMRM**/**TMRE**, **GR555-mito** and **GR650-mito** are not fast-responsive MMP indicators due to their increased lipophilicity.^46−48^ Overall, rhodamine-COT-based mitochondrial dyes supplement their cyanine counterparts (such as PK Mito dyes^11,22^) for structural imaging of mitochondria.

### Fluorogenic COT-Rhodamines Exhibit Lower Phototoxicity for General Organelle Imaging

The emerging class of fluorogenic rhodamines, bearing a dynamic equilibrium between a fluorescent zwitterion and a nonfluorescent spirolactone/lactam form, have enabled wash-free imaging of various organelles.^29^ We speculated that the COT-conjugation of rhodamines can be integrated into the spirocyclization motif, in which a COT-sulfonamide group (Supplementary Scheme S4, see Synthesis Method for details) instead of a COT-methanol is introduced to the 3-carboxy position, making it a cell-permeable and fluorogenic rhodamine core.

**Figure 3:**
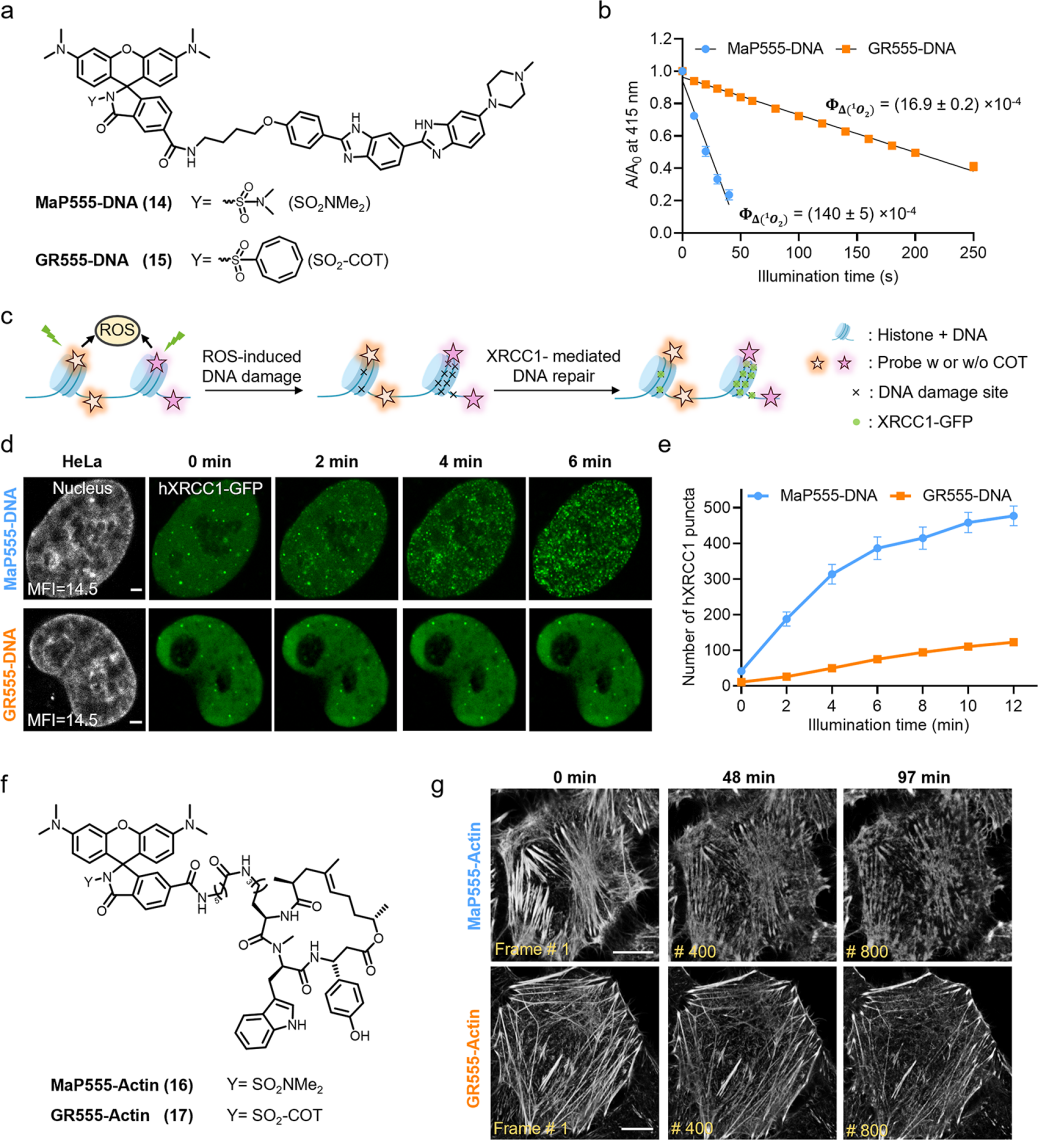
**Targetable gentle rhodamines for live-cell imaging of DNA and cytoskeleton.** (a) Chemical structures of **MaP555-DNA** (**14**, blue) and **GR555-DNA** (**15**, orange). (b) *In vitro* singlet oxygen generation experiments of **MaP555-DNA** and **GR555-DNA**. The maximum absorption of DPBF at 415 nm was measured under continuous irradiation with a 520–530 nm LED lamp in the presence of each dye (absorbance at 525 nm = 0.15; concentrations: **14**, 1 μM; **15**, 1.1 μM) in air-saturated acetonitrile containing 0.1% TFA. The absolute singlet oxygen quantum yields (Φ_Δ_) are given on the graph. Data points represent averaged and normalized DPBF decay curves of three independent repeats. Error bars indicate the standard deviation. (c) Schematic representation of the DNA damage assay based on hXRCC1-GFP. Upon (light-induced) DNA damage, hXRCC1-GFP gets recruited to the damaged site. (d) Live cell confocal images of HeLa cells expressing hXRCC1-GFP at a frame rate of 2 min/frame. HeLa cells were labeled with **MaP555-DNA** (gray, 200 nM) or **GR555-DNA** (gray, 2 μM) for 60 min at 37 °C. Puncta formation in the time-lapse images of DNA repair protein hXRCC1 fused with GFP indicates the DNA damage level (green). Scale bars = 2 μm. (e) Semiquantitative analysis of cellular phototoxicity of **MaP555-DNA** and **GR555-DNA** of HeLa hXRCC1-GFP cells, as measured by the total number of hXRCC1-GFP puncta from the experiments shown in **d**. Data points represent the averaged hXRCC1-GFP number of 11 cells from five independent experiments. Error bars indicate the standard error of the mean. (f) Chemical structures of the actin dyes **MaP555-Actin** (**16**, blue) and **GR555-Actin** (**17**, orange). (g) Long-term time-lapse confocal recordings of HeLa cells at a frame rate of 7.27 s/frame. HeLa cells were labeled with **MaP555-Actin** or **GR555-Actin** (both 100 nM with 10 μM verapamil) for 3 h at 37 °C. Cells labeled with **GR555-Actin** showed no shrinkage and fracture of actin filaments during the time of recording. Scale bars = 10 μm.

We first derivatized the spirocyclic TMR-COT conjugate with Hoechst conjugated at the 5-carboxyl position, yielding **GR555-DNA** (**15**) (Figure 3a). **GR555-DNA** exhibited an 8-fold fluorescent intensity increase (“turn-on”) upon binding to hairpin-DNA (hpDNA) *in vitro*, exhibiting a higher fluorogenicity than **MaP555-DNA** and a comparable fluorescence quantum yield (Φ_MaP555-DNA_ = 0.35 and Φ_GR555-DNA_ = 0.37 after binding to DNA, Supplementary Table S3). Compared to the parent compound **MaP555-DNA** (**14**), the COT-derived counterpart exhibited 8-fold lower singlet oxygen generation under a 520–530 nm LED illumination (Figure 3b and Table S1).

For live-cell staining, **GR555-DNA** showed nuclear specificity. The new DNA stain displayed a lower cell permeability than **MaP555-DNA**, presumably due to a larger molecular weight. Yet for HeLa cell staining, labeling with 2 μM **GR555-DNA** or 0.2 μM **MaP555-DNA** for 60 min resulted in similar brightness and signal-to-noise ratios under no-wash conditions (Supplementary Figure S13a). Notably, the staining conditions of **GR555-DNA**, although slightly more demanding, did not lead to significant cytotoxicity (Supplementary Figure S14). We then exploited a DNA repair imaging assay to semiquantitatively characterize the phototoxicity of DNA dyes in live cells (schematic diagram shown in Figure 3c). X-ray repair cross-complementing protein 1 (XRCC1) is a scaffolding protein that accumulates at sites of DNA-damage and recruits other proteins involved in DNA repair pathways.^49^ hXRCC1-GFP is evenly distributed in the nucleus of healthy cells, while upon DNA damage it gets recruited to the damaged site and exhibits multiple fluorescent puncta patterns in the nucleus, giving a sensitive assay of DNA damage under stress.^50^ HeLa cells expressing hXRCC1-GFP labeled with **MaP555-DNA** showed a gradual increase in the number of hXRCC1-GFP puncta after 2 min-exposure to a 560 nm pulse laser, and the puncta numbers plateaued after 10 min-exposure. In contrast, HeLa cells labeled with **GR555-DNA** had a lower number of hXRCC1-GFP puncta than those treated with **MaP555-DNA** (Figure 3d, e). To control the photobleaching factors, we also compared the photostability of the **GR555-DNA** and **MaP555-DNA** in living cells. Neither of the two DNA dyes showed significant fluorescence intensity decay after 12 min of exposure to the 560 nm pulsed laser (Supplementary Figure S13b). Therefore, we concluded that the COT-conjugation strategy reduced the cellular phototoxicity when such fluorophore is attached to a DNA-targeting moiety and ultimately minimized the DNA damage in live-cell microscopy.

Next, we aimed to develop a low-phototoxicity probe for imaging of the cytoskeleton in live cells. We conjugated **GR555** to a Jasplakinolide derivative binding to F-actin,^26^ yielding **GR555-Actin** (compound **17**, Figure 3f). **GR555-Actin** has a ∼6-fold reduced singlet oxygen generation compared to the reference dye **MaP555-Actin** (compound **16**, Supplementary Figure S16 and Table S1). In long-term time-lapse confocal imaging, **GR555-Actin** exhibited reduced phototoxicity and enhanced brightness and photostability (Supplementary Figure S13). Actin filaments of HeLa cells stained with **MaP555-Actin** tended to shrink, accompanied by a large attenuation of the fluorescence intensity, and gradually disintegrated and fractured into short strands after 41–47 min (350–400 frames). In comparison, **GR555-Actin** enabled acquisition of 118 min (1000 frames) with integral actin filament structures (Figure 3g and Supplementary Movie S1). Together, these data demonstrate that the COT-conjugated actin probe enables long-term imaging of cellular structures with low phototoxicity.

### Fluorogenic and Gentle COT-Rhodamines for HaloTag

**Figure 4:**
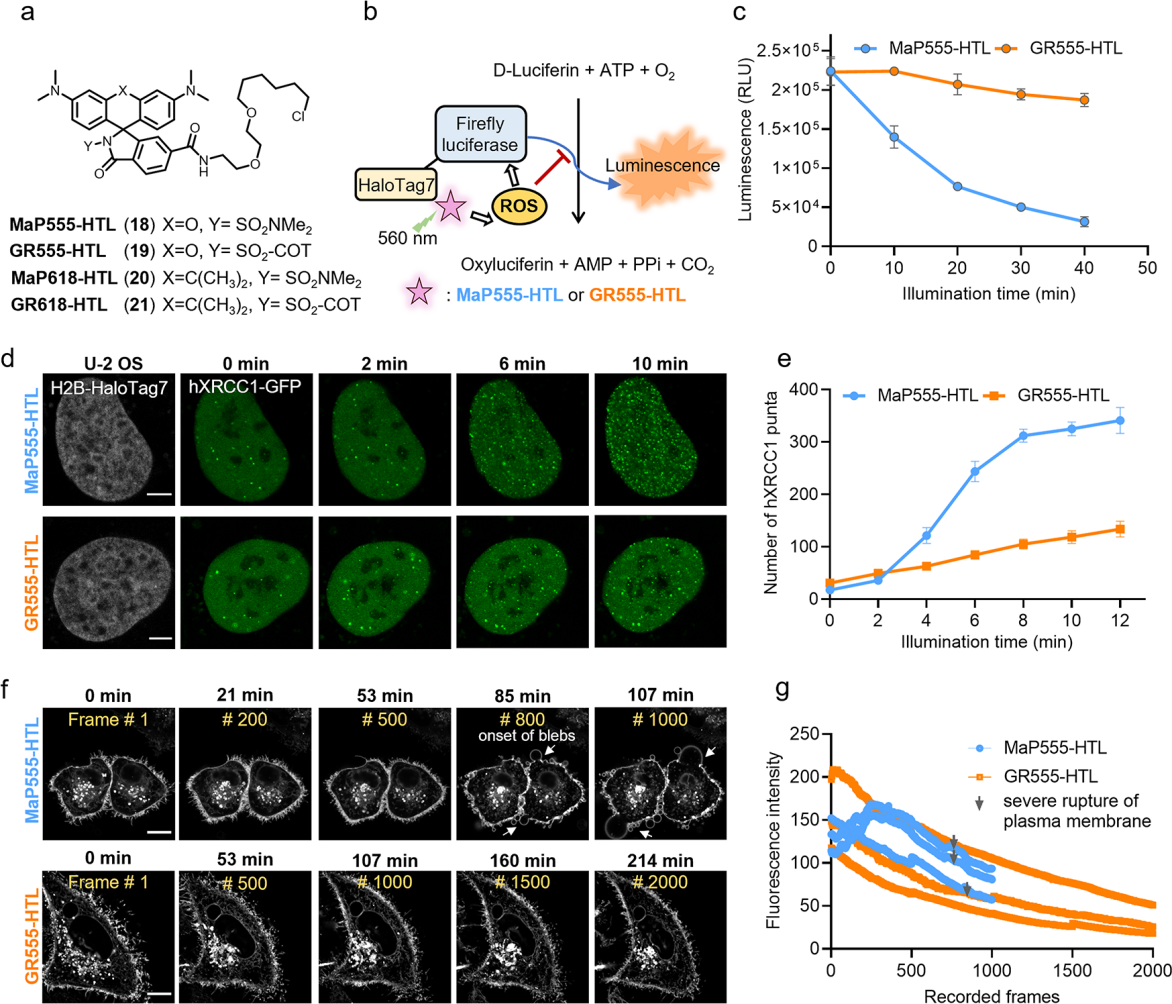
**Gentle rhodamines for live-cell imaging of cellular proteins using self-labeling protein tags.** (a) Chemical structures of **MaP555/618**, **GR555/618** (**18**–**21**) derivatives coupled to the HaloTag Ligand (chloroalkane substrate). (b) Schematic diagram of the protein damage assay. Firefly luciferase-HaloTag7 (FLuc-HaloTag7) is labeled with **MaP555-HTL** and **GR555-HTL** and the photodamage under long-term illumination assessed by the luminescence generated by FLuc afterward. (c) Light-induced photodamage of FLuc-HaloTag7 labeled with **MaP555-HTL** and **GR555-HTL***in vitro*. The fully labeled protein was illuminated and after different time points the protein damage was assessed by D-Luciferin addition and luminescence measurements. Data points represent the averaged luminescence of three independent experiments. Error bars indicate the standard error of the mean. (d) Live-cell confocal recordings (gray) of U-2 OS cells expressing H2B-HaloTag7 (stable) and DNA repair protein hXRCC1-GFP (transient) at a frame rate of 2 min/frame. U-2 OS cells were labeled with **MaP555-HTL** or **GR555-HTL** (both 500 nM) for 30 min at 37 °C. Scale bars: 5 μm. (e) Semiquantitative analysis of cellular phototoxicity analysis of **GR555-HTL** and **MaP555-HTL** of U-2 OS H2B-HaloTag7 cells, as measured by counting the total number of hXRCC1-GFP puncta. Data points represent the averaged hXRCC1-GFP number of five cells from five independent experiments. Error bars indicate the standard error of the mean. (f) Long-term time-lapse confocal recordings of HeLa cells expressing HaloTag7-PDGFR^tmb^ at a frame rate of 6.41 s/frame. HeLa cells were labeled with **MaP555-HTL** or **GR555-HTL** (both 500 nM) for 30 min at 37 °C. Cells labeled with **GR555-HTL** showed no appearance of blebs and intact plasma membrane for the time of recording. Scale bars = 10 μm. (g) Photobleaching curves of HeLa cells expressing HaloTag7-PDGFR^tmb^ labeled with **MaP555-HTL** or **GR555-HTL** under continuous time-lapse confocal recordings using a 561 nm pulsed laser. The gray arrows indicate the onset of blebbing and membrane disruption. Each curve represents the bleaching curve of an individual HeLa cell.

Self-labeling protein (SLP) tags enable selective labeling of cellular proteins. In combination with cell-permeable fluorophores, SLPs are advantageous over fluorescent proteins (FPs) because they offer the means to select between bright fluorescent probes of different colors and spectroscopic properties, making them the prime method for live-cell nanoscopy. However, the dyes attached to the tags are exposed to the cellular environment and therefore potentially more phototoxic. In contrast, the chromophores of FPs are shielded to insulate the sensitization process in ROS generation.^51^ To address the phototoxicity of SLP substrates, we coupled our gentle spirocyclic rhodamine dye to the chloroalkane HaloTag Ligand (HTL) to obtain **GR555-HTL** (**19**) (Figure 4a). In addition, to broaden the spectral range and explore a different fluorophore scaffold, we synthesized the corresponding red-shifted carborhodamine derivative **GR618-HTL** (**21**), as an analog to its previously published parent dye **MaP618-HTL** (**20**)^29^ (Figure 4a).

We compared the propensity of both probes to exist in the spirocyclic form by water-dioxane titrations: For **GR555-HTL**, the spirocyclization equilibrium lies more toward the fluorescent zwitterionic form (D_50_: 40) compared to **MaP555-HTL** (**18**) (D_50_: 66), yet it displays a mild (∼4-fold) fluorescence “turn-on” and similar brightness when bound to HaloTag7 (Supplementary Table S3 and Figure S17a,b). **GR618-HTL** predominantly adopts the colorless spirolactam form (D_50_ > 75) and displays a great fluorogenic potential by its ∼41-fold fluorescence “turn-on” upon HaloTag7 labeling (Supplementary Table S3 and Figure S17c,d), which makes it attractive for no-wash, live-cell applications. Despite **GR618-HTL**’s slower labeling kinetics of HaloTag7 proteins compared to **MaP618-HTL** (Supplementary Figure S18), both **GR-HTL** probes displayed equivalent signal brightness and fluorescence lifetimes compared to their “MaP” analogs in live cells (Supplementary Figure S19, Table S3). Also, the DPBF assay demonstrated both **GR555-HTL** and **GR618-HTL** featured lower singlet oxygen generation (by 10-/4-fold respectively) than their “MaP” counterparts (Supplementary Figure S20 and Table S1).

We then designed an *in vitro* assay using Firefly luciferase (FLuc) HaloTag7-fusion protein labeled with **GR555-HTL** or **MaP555-HTL** to assess the ROS-induced protein damage (Figure 4b and Supplementary Figure S21). After green light excitation for up to 40 min, D-Luciferin was added and the luciferase activity was measured using a bioluminescence assay. FLuc-HaloTag7 labeled with **MaP555-HTL** exhibited a severe drop (>85%) of enzymatic activity during the 40 min illumination, indicating that the ROS generated from **MaP555-HTL** was profoundly damaging the FLuc. In contrast, FLuc-HaloTag7 labeled with **GR555-HTL** showed only a drop of <20% after 40 min of illumination. Of note, the remaining fluorescence signal after 40 min illumination of protein samples was >60%, in which **GR555-HaloTag** was slightly more photostable than **MaP555-HaloTag** (Figure 4c and Supplementary Figure S21d). Therefore, we conclude that the lower phototoxicity attributed to **GR555-HTL** indeed prevents damage to closeby proteins as compared to regular **MaP555-HTL** labeling.

To further validate the reduced phototoxicity of **GR555-HTL** at the cellular level, we fused HaloTag7 to the nuclear histone 2B (H2B), a key chromatin component closely associated with DNA and assessed phototoxicity using XRCC1 assay (Figure 3c). U-2 OS cells expressing H2B-HaloTag7 were stained with **GR555-HTL** or **MaP555-HTL**, yielding comparable fluorescence signals in the nucleus (Supplementary Figures S19 and S22). During 560 nm laser exposure, **MaP555-HTL** samples showed a rapid increase in hXRCC1-GFP puncta with a maximum puncta number of about 300–400, indicating significant DNA damage. In contrast, the cells labeled with **GR555-HTL** experienced a slow increase in hXRCC1-GFP puncta with a maximum puncta number of ∼100 (Figure 4d,e), supporting that COT conjugation significantly reduces cellular photodamage (by 3- to 4-fold) on H2B-HaloTag7 under long-term illumination. Similar results were obtained for **GR618-HTL** (Supplementary Figure S23). Furthermore, long-term confocal imaging of plasma membrane-targeted HaloTag 7 revealed **MaP555-HTL** caused membrane damage with severe rupture of plasma membrane and formation of blebs after 85 min (800 frames), while **GR555-HTL**-labeled cells did not undergo significant apoptosis during up to 214 min (2000 frames) during up to 214 min (2000 frames) (Figure 4f,g and Supplementary Movie S2). HeLa cells expressing HaloTag7 on the plasma membrane exhibited better cell viability when labeled with Gentle Rhodamine HaloTag7 probes compared to their traditional counterparts (Figure 4f and Supplementary Figure S24), endorsing GR-HTL as superior ligands for prolonged recordings.

**Figure 5:**
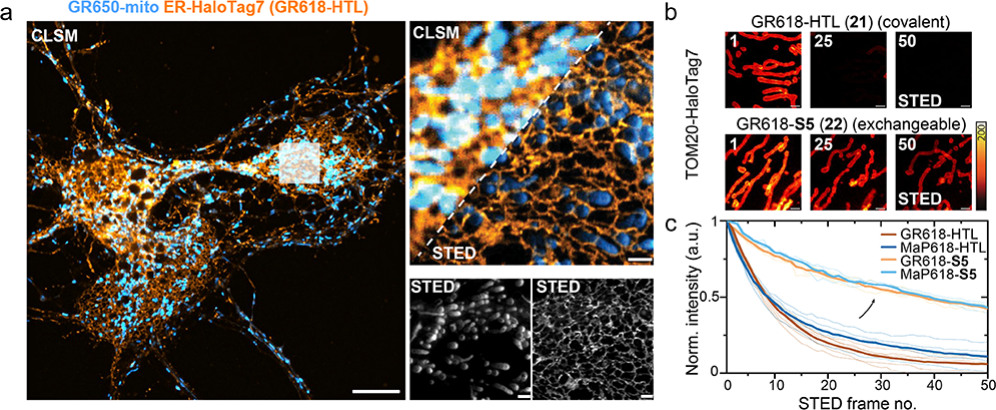
**Gentle rhodamines are privileged dyes for long-term multicolor STED nanoscopy recordings.** (a) Dual-color confocal laser-scanning microscopy (CLSM) and STED imaging of the ER and mitochondria using gentle rhodamine probes in live cultured rat hippocampal neurons. Neurons (10 DIV) expressing CalR-HaloTag7 (rAAV transduction) were stained with **GR618-HTL** (endoplasmic reticulum, cyan, 500 nM) and **GR650-mito** (mitochondria, red, 50 nM) for 30 min at 37 °C. **GR618-HTL** was excited with a 561 nm laser and **GR650-mito** with a 640 nm laser. Both dyes were depleted with a 775 nm depletion laser (STED). White rectangle in the CLSM overview (right) shows magnified FOV for STED imaging. Scale bars = 10 μm (overview), 2 μm (magnification). (b) Time-lapse STED imaging showcasing different photobleaching behavior of **MaP/GR618** covalently conjugated to HaloTag (HTL) or its exchangeable counterpart (S5). Multiframe STED imaging of U-2 OS mitochondria outer membrane (TOM20-HaloTag7) labeled with **GR618-(x) HTLs** over 50 consecutive frames in a 10 × 10 μm ROI using **MaP618/GR618-HTL**, **-S5**. Frame numbers are indicated in the top left corner. Scale bars: 1 μm. (c) Bleaching curves (thick lines: mean value, thin lines: individual experiments) plotted for at least 4 image series (*n* ≥ 4) as shown in (b).

The red dye **GR618-HTL** opens new possibilities for spectral multiplexing and super-resolution imaging with low phototoxicity and excellent photostability. First, it can be combined with orange and near-infrared probes for multicolor imaging of several cellular compartments: We fused HaloTag7 to the calreticulin protein and a KDEL signal sequence (CalR-HaloTag7-KDEL) in order to target it to the endoplasmic reticulum (ER). In this way, the ER can be labeled with **GR618-HTL** and imaged together with DNA (**GR555-DNA**) and mitochondria (**GR650-mito**) in primary rat hippocampal neurons with minimal phototoxicity over 4 h (150 frames) (Supplementary Figure S25 and Movie S3). Second, **GR618-HTL** is compatible with 775 nm depletion lasers in STED systems, enabling dual-color imaging of mitochondria and the ER in live neurons (Figure 5a). For time-lapse STED imaging, it has been shown that exchangeable fluorophore labels boost the photostability due to the replacement of bleached probes, which is not possible with covalent labeling approaches.^52^ We synergistically combined the GR strategy with exchangeable HaloTag7 ligands (xHTLs).^53^**GR618** was conjugated to an xHTL linker, giving rise to the noncovalent probes **GR618-S5** (**22**) (Supplementary Figure S26a). The photobleaching behaviors of covalent (**MaP/GR618-HTL**) and the exchangeable HaloTag substrates (**MaP/GR618-S5**) were profiled using time-lapse STED nanoscopy on U-2 OS cells expressing HaloTag7 fused to an outer mitochondrial membrane marker protein (TOM20, Figure 5b). Here, **GR618-S5** exhibits a slower photobleaching rate compared to **GR618-HTL**, suggesting the compatibility of **GR618** with xHTLs which gives approximately 5-fold enhancement in photostability (Figure 5c). This finding was confirmed with a second xHTL (Hy5, Supplementary Figure S26). Notably, the photobleaching profiles of **MaP618** and **GR618** are largely the same. These data, along with the similar photobleaching profiles of **GR555** and **MaP555** (Figure 4g), suggest that the COT conjugation alone is not able to improve the photostability of HaloTag-labeled rhodamines.

**Figure 6:**
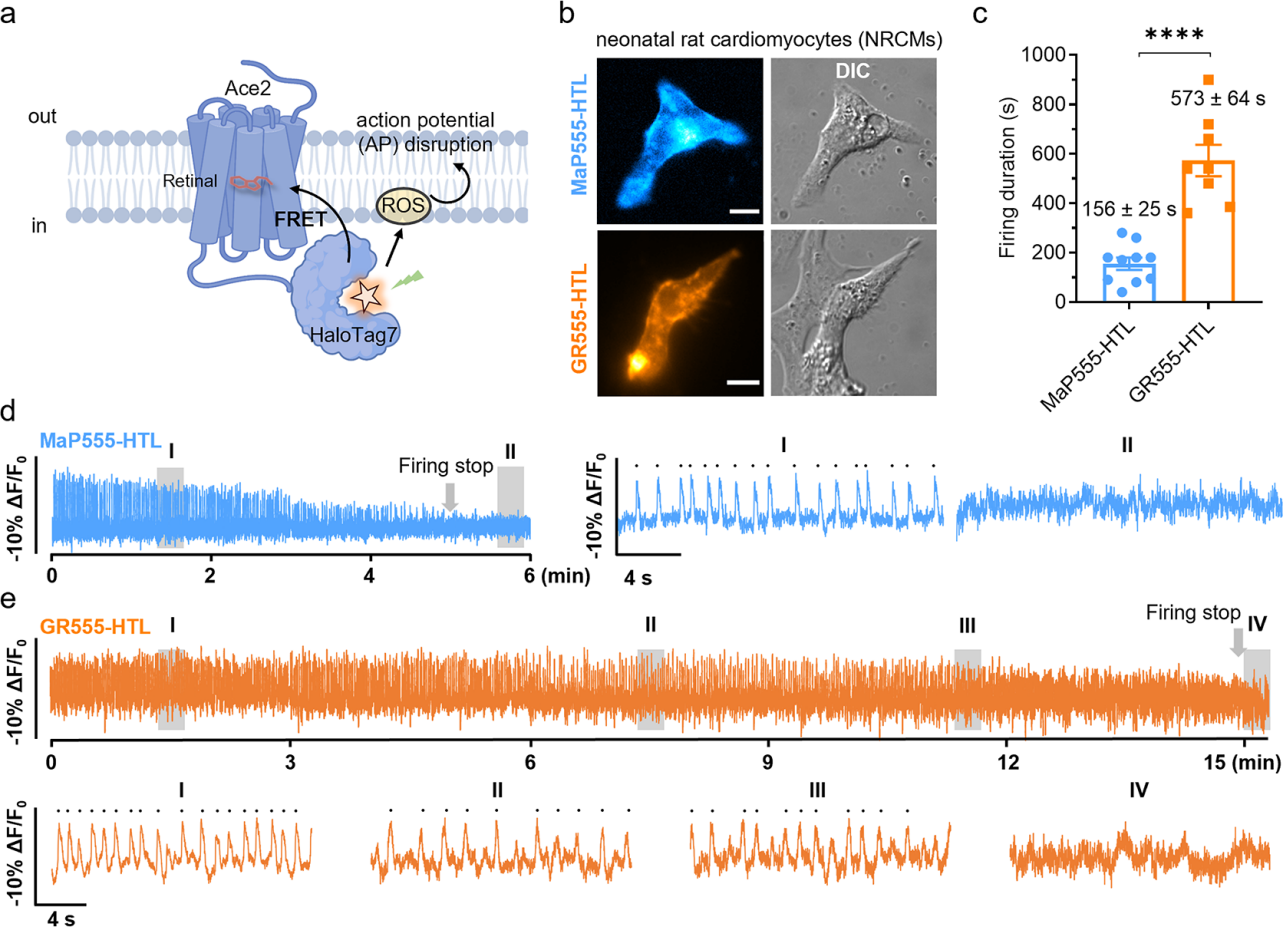
**Gentle rhodamines are privileged dyes for voltage imaging of primary cells.** (a) Schematic representation of phototoxicity during long-term voltage imaging based on chemigenetic voltage-indicator Voltron. (b) Wide-field microscopy of the neonatal rat cardiomyocytes (NRCMs) expressing Voltron (Ace2-HaloTag7) and labeled with **MaP555-HTL** or **GR555-HTL** (both 100 nM) for 25 min at 37 °C. Scale bar = 10 μm. (c) Firing duration of NRCMs expressing Voltron (Ace2-HaloTag7) labeled with **MaP555-HTL** or **GR555-HTL**. The illumination intensities of 561 nm lasers were 2.16 W·cm^–2^. Bars indicate the mean of seven cells. Error bars indicate the standard error of the mean. Significance was determined using a two-tailed unpaired *t* test followed by Sidak’s multiple comparisons test. *P* = **** < 1.0 × 10^–4^. (d,e) A representative fluorescence trace of NRCMs expressing Voltron and labeled with **MaP555-HTL** or **GR555-HTL**. Each peak on the traces showed spontaneous spikes of each NRCM and signals were corrected for photobleaching. The illumination intensity of the 561 nm laser was 2.16 W/cm^2^. (d) 6 min (36,000 frames) recordings at 100 frames/sec were performed. Two zoomed-in signals (i–ii) from two shaded regions (I–II) were presented at the right. Each black dot represents one spontaneous spike. (e) 15 min (90,000 frames) recordings at 100 frames/sec were performed. Four zoomed-in signals (i–iv) from four shaded regions (I–IV) were presented at the bottom. Each black dot represents one spontaneous spike.

Finally, we showcase long time-lapse functional imaging on primary cells by combining **GR-HTL** probes with a chemigenetic voltage indicator (Voltron), which consists of genetically encoded Ace2 rhodopsin fused to HaloTag7^54^ and offers a brighter signal and a larger dynamic range^55^ than FRET-based indicators employing fluorescent proteins. However, the use of Voltron can result in phototoxicity, hampering voltage recordings (Figure 6a). We labeled neonatal rat cardiomyocytes (NRCMs) expressing Voltron with **GR555-HTL** or **MaP555-HTL** respectively (Figure 6b) and monitored their activity by recording the changes in fluorescence (ΔF/F_0_) to trace their spontaneous electrical signals. However, after 156 ± 25 s of continuous imaging at 100 Hz, the cardiomyocytes labeled with **MaP555-HTL** stopped beating and firing due to accumulated phototoxicity (Figure 6c,d). In contrast, cardiomyocytes labeled with **GR555-HTL** provided a continuous voltage signal for up to 573 ± 64 s under identical imaging conditions (561 nm laser illumination at 2.16 W/cm^2^) before the firing stopped (Figure 6c,e). In addition, the alteration in AP morphology was more pronounced when imaging with **MaP555-HTL** than that with **GR555-HTL**, giving a vanishing signal in 6 min (Supplementary Figure S27). This highlights the advantage of gentle rhodamines in reducing phototoxicity for long-term physiological studies in primary cells.

## Discussion and Conclusions

With the increasing demand for spatial and temporal resolution in live-cell imaging, we argue that phototoxicity in live-cell imaging is a fundamental challenge of growing importance.^5,7^ In this work, we demonstrate that rhodamine dyes, the privileged toolkit for live cell imaging, can be rendered less phototoxic upon the conjugation of COT at the proximal 3-carboxyl group. Furthermore, photodamage was thoroughly assessed through assays ranging from *in vitro* ROS generation, proximity protein damage, cell death, and morphological and physiological alterations. It is noteworthy that our demonstrated assays on phototoxicity are by no means exhaustive, as our understanding of the biology of ROS is still preliminary.^7,56^ In this sense, this work would hopefully inspire further studies along this line.

We have now established TSQ conjugation as a primary approach to systematically alleviate phototoxicity^11,22^ while minimizing the nonspecific binding of dyes is another viable direction in parallel.^57^ In the future, we will further assess and optimize the tissue permeability of gentle rhodamines toward *in vivo* applications. As rhodamines are modular building blocks that can be readily combined with state-of-the-art labeling technologies, the gentle rhodamines reported here thus represent chemical solutions to phototoxicity issues in live-cell imaging. These chemical approaches would eventually synergize with mathematical, optical, and spectroscopical approaches^1,58^ to enable time-lapse dynamic imaging, offering long-lasting fluorescence signals that transfer into multiplexed spatial and temporal information with uncompromised physiological relevance.

## Data Availability

All data reported in this paper will be shared by the corresponding author upon reasonable request.

## Supporting Information Available

Description of all synthetic procedures, chemical characterization of synthesized compounds, material methods for biological and imaging assays, and supplementary figures for chemical characterization and cell-based assays (PDF)
Movie S1. Long-term time-lapse confocal recordings of HeLa cells labeled with MaP555-Actin or GR555-Actin (MP4)
Movie S2. Long-term time-lapse confocal recordings of HeLa HaloTag7-PDGFR^tmb^ cells labeled with MaP555-HTL or GR555-HTL (MP4)
Movie S3. Three-color time-lapse confocal recordings of mitochondria, endoplasmic reticulum, and DNA on rat hippocampal neurons expressing CalR-HaloTag7-KDEL (MP4)
